# A population-based method to determine the time-integrated activity in molecular radiotherapy

**DOI:** 10.1186/s40658-021-00427-x

**Published:** 2021-12-14

**Authors:** Deni Hardiansyah, Ade Riana, Peter Kletting, Nouran R. R. Zaid, Matthias Eiber, Supriyanto A. Pawiro, Ambros J. Beer, Gerhard Glatting

**Affiliations:** 1 Medical Physics and Biophysics Division, Physics Department, Faculty of Mathematics and Natural Sciences, Universitas Indonesia, 16424 Depok, Indonesia; 2 Medical Radiation Physics, Department of Nuclear Medicine, Ulm University, Albert-Einstein-Allee 23, 89081 Ulm, Germany; 3 Department of Nuclear Medicine, Ulm University, 89081 Ulm, Germany; 4 Department of Nuclear Medicine, Klinikum rechts der Isar, Technische Universität München, 81675 Munich, Germany

**Keywords:** TIAs, Absorbed dose, Model selection

## Abstract

**Background:**

The calculation of time-integrated activities (TIAs) for tumours and organs is required for dosimetry in molecular radiotherapy. The accuracy of the calculated TIAs is highly dependent on the chosen fit function. Selection of an adequate function is therefore of high importance. However, model (i.e. function) selection works more accurately when more biokinetic data are available than are usually obtained in a single patient. In this retrospective analysis, we therefore developed a method for population-based model selection that can be used for the determination of individual time-integrated activities (TIAs). The method is demonstrated at an example of [^177^Lu]Lu-PSMA-I&T kidneys biokinetics. It is based on population fitting and is specifically advantageous for cases with a low number of available biokinetic data per patient.

**Methods:**

Renal biokinetics of [^177^Lu]Lu-PSMA-I&T from thirteen patients with metastatic castration-resistant prostate cancer acquired by planar imaging were used. Twenty exponential functions were derived from various parameterizations of mono- and bi-exponential functions. The parameters of the functions were fitted (with different combinations of shared and individual parameters) to the biokinetic data of all patients. The goodness of fits were assumed as acceptable based on visual inspection of the fitted curves and coefficients of variation CVs < 50%. The Akaike weight (based on the corrected Akaike Information Criterion) was used to select the fit function most supported by the data from the set of functions with acceptable goodness of fit.

**Results:**

The function $A_{1} { }\beta { }e^{{ - \left( {\lambda_{1} + \lambda_{{{\text{phys}}}} } \right)t}} + A_{1} { }\left( {1 - \beta } \right){ }e^{{ - \left( {\lambda_{{{\text{phys}}}} } \right)t}}$ with shared parameter $\beta$ was selected as the function most supported by the data with an Akaike weight of 97%. Parameters $A_{1}$ and $\lambda_{1}$ were fitted individually for every patient while parameter $\beta { }$ was fitted as a shared parameter in the population yielding a value of 0.9632 ± 0.0037.

**Conclusions:**

The presented population-based model selection allows for a higher number of parameters of investigated fit functions which leads to better fits. It also reduces the uncertainty of the obtained Akaike weights and the selected best fit function based on them. The use of the population-determined shared parameter for future patients allows the fitting of more appropriate functions also for patients for whom only a low number of individual data are available.

## Background

Individual treatment planning is desirable for radionuclide therapy to maximize tumour absorbed dose while sparing organs at risk [1–3]. The absorbed doses are determined for the largest part by the time-integrated activities (TIAs) [4, 5]. The TIAs are equal to the number of disintegrations of the used radionuclide in the considered organ. To calculate the TIAs, a mathematical function is first fitted to the measured biokinetic data obtained from 2D or 3D imaging at multiple time points [6–9], and this function is then integrated from time zero to infinity. The calculated TIA values based on this fitting method depend on the chosen fit function [10]. Therefore, using the “optimal” fit function [11] is crucial for the accurate and precise determination of the TIAs and subsequently the absorbed doses. Relevant criteria for an optimal fit function are that.

1. the investigated function fits the data, i.e. the goodness of fit is satisfactory, and
2. the function is most supported by the observed data. "Most" here refers to a set of reasonable functions defined by the investigator.

While item (1) can be easily checked by applying standard criteria such as visual inspection of the fitted graph, quantitative assessment using coefficient of variations of the fitted parameters (< 50%) and the constraints for the correlation matrix elements (absolute values being lower than 0.8) [8], item (2) requires model (or function) selection based on quantitative analysis of the corrected Akaike information criterion (AICc) [11, 12].

Model selection has two inputs: On the one hand the set of models and on the other hand the underlying observed data. The former, however, depends on the latter, as few data only allow the use of models (or corresponding functions) with few parameters.

In nuclear medicine, the measurement of biokinetics is often only carried out at a few time points. Therefore, instead of using the data of only a single patient, i.e. individual-based model selection (IBMS), including the data of additional patients with the same disease treated with the same radiopharmaceutical might be important to determine an optimal fit function (item (2) above). Such a population-based model selection (PBMS) increases the ratio of number of observed data used as input to the number of estimated parameters and thus reduces the uncertainty in the model selection. Moreover, it allows to use an expanded model set, as functions with a higher number of parameters become possible. In addition, information about the functional shape of the time-activity curve of previous patients might be used for future patients.

In this work, we therefore present a general method to improve the calculation of TIAs using biokinetic data of a population instead of a single patient only. The method performs the required model selection based on a PBMS approach and is presented at the example of kidneys biokinetics in [^177^Lu]Lu-PSMA-I&T radioligand therapy. For this purpose, a set of mathematical models or functions is defined, a population-based fit is performed and the function most supported by the data is selected using the Akaike weights method. The developed method can be used to determine individual TIAs of future patients using the best function obtained from a previously measured population.

## Material and methods

### Biokinetic data of [^177^Lu]Lu-PSMA-I&T in kidneys

Thirteen patients with metastatic castration-resistant prostate cancer were included in this retrospective analysis [13, 14]. All patients underwent [^177^Lu]Lu-PSMA-I&T radioligand therapy (RLT) and post-therapeutic planar whole-body scintigraphies. The biokinetic data (the time-activity data) of [^177^Lu]Lu-PSMA-I&T RLT in kidneys were calculated from the kidneys regions of interest using the geometric mean of anterior and posterior counts with background corrections. From thirteen patients, 3 patients had 5 time points data, 1 patient had 4 time points data and 9 patients had 3 time points data. The biokinetic data were obtained at (1.1 ± 0.7) h, (20.7 ± 2.3) h, (51.0 ± 10.1) h, (92.3 ± 47.2) h, (163.8 ± 2.1) h p.i..

### Investigated set of exponential functions

Sums of exponential functions with increasing complexity were used in the investigated model set, as such mathematical functions are commonly used to describe biological processes [6–9]:

1 $$f_{2a} \left( t \right) = 100 e^{{ - \left( {\lambda_{1} + \lambda_{{{\text{phys}}}} } \right)t}} - 100 e^{{ - \left( {\lambda_{2} + \lambda_{{{\text{phys}}}} } \right)t}}$$

2 $$f_{2b} \left( t \right) = A_{1} e^{{ - \left( {\lambda_{1} + \lambda_{{{\text{phys}}}} } \right)t}}$$

3 $$f_{2c} \left( t \right) = A_{1} e^{{ - \left( {\lambda_{1} + \lambda_{{{\text{phys}}}} } \right)t}} - A_{1} e^{{ - \left( {\lambda_{{{\text{phys}}}} } \right)t}}$$

4 $$f_{2d} \left( t \right) = - A_{1} e^{{ - \left( {\lambda_{1} + \lambda_{{{\text{phys}}}} } \right)t}} + A_{1} e^{{ - \left( {\lambda_{{{\text{phys}}}} } \right)t}}$$

5 $$f_{2e} \left( t \right) = A_{1} e^{{ - \left( {\lambda_{1} + \lambda_{{{\text{phys}}}} } \right)t}} + (100 - A_{1} ) e^{{ - \left( {\lambda_{{{\text{phys}}}} } \right)t}}$$

6 $$f_{3a} \left( t \right) = A_{1} e^{{ - \left( {\lambda_{1} + \lambda_{{{\text{phys}}}} } \right)t}} + A_{2} e^{{ - \left( {\lambda_{{{\text{phys}}}} } \right)t}}$$

7 $$f_{3b} \left( t \right) = A_{1} e^{{ - \left( {\lambda_{1} + \lambda_{{{\text{phys}}}} } \right)t}} - A_{1} e^{{ - \left( {\lambda_{2} + \lambda_{{{\text{phys}}}} } \right)t}}$$

8 $$f_{3c} \left( t \right) = A_{1} e^{{ - \left( {\lambda_{1} + \lambda_{{{\text{phys}}}} } \right)t}} + \left( {100 - A_{1} } \right) e^{{ - \left( {\lambda_{2} + \lambda_{{{\text{phys}}}} } \right)t}}$$

where $f_{ia}$ is a fit function with $i$ parameters, the $A_{i} \ge 0$ are the prefactors, $\lambda_{{{\text{phys}}}}$ is the physical decay constant of the radionuclide calculated from the half-life $T_{1/2}$ of ^177^Lu $\left( {\lambda_{{{\text{phys}}}} = \ln \left( 2 \right)/T_{1/2} } \right)$ and $\lambda_{1}$ and $\lambda_{2}$ describe the biological clearance rates of the radiopharmaceutical. In addition, the following functions were also used which were defined in analogy to the case of degenerate eigenvalues for a damped oscillator (note the additional factor *t*):

9 $$f_{3d} \left( t \right) = A_{1} t e^{{ - \left( {\lambda_{1} + \lambda_{{{\text{phys}}}} } \right)t}} + A_{2} e^{{ - \left( {\lambda_{1} + \lambda_{{{\text{phys}}}} } \right)t}}$$

10 $$f_{2a,3d} \left( t \right) = A_{1} t e^{{ - \left( {\lambda_{{{\text{phys}}}} } \right)t}} + A_{2} e^{{ - \left( {\lambda_{{{\text{phys}}}} } \right)t}}$$

11 $$f_{2b,3d} \left( t \right) = A_{1} t e^{{ - \left( {\lambda_{1} + \lambda_{{{\text{phys}}}} } \right)t}}$$

12 $$f_{2c,3d} \left( t \right) = A_{1} t e^{{ - \left( {\lambda_{1} + \lambda_{{{\text{phys}}}} } \right)t}} + 100 e^{{ - \left( {\lambda_{1} + \lambda_{{{\text{phys}}}} } \right)t}}$$

The three functions (10)–(12) are derived from Eq. (9) by reducing the number of fit parameters. In addition to the functions in Eqs. (1)–(12), we examined the functions below using all biokinetic data of the patient population and a shared parameter approach. The shared parameters are assumed to be the same for all patients and are estimated for all data in the patient population together. The other parameters were individually estimated from the data. All the following functions are derived from function $f_{3a}$ (Eq. (6)) with different shared parameters (Eqs. (13)–(15)) and different parameterizations (Eqs. (16)–(18)):

13 $$f_{3aS1} \left( t \right) = A_{1} e^{{ - \left( {\lambda_{1} + \lambda_{{{\text{phys}}}} } \right)t}} + A_{2} e^{{ - \left( {\lambda_{{{\text{phys}}}} } \right)t}} \;{\text{with}}\,{\text{shared}}\,{\text{parameter}}\;A_{1}$$

14 $$f_{3aS2} \left( t \right) = A_{1} e^{{ - \left( {\lambda_{1} + \lambda_{{{\text{phys}}}} } \right)t}} + A_{2} e^{{ - \left( {\lambda_{{{\text{phys}}}} } \right)t}} \;{\text{with}}\,{\text{shared}}\,{\text{parameter}}\;\lambda_{1}$$

15 $$f_{3aS3} \left( t \right) = A_{1} e^{{ - \left( {\lambda_{1} + \lambda_{{{\text{phys}}}} } \right)t}} + A_{2} e^{{ - \left( {\lambda_{{{\text{phys}}}} } \right)t}} \;{\text{with}}\,{\text{shared}}\,{\text{parameter}}\;A_{2}$$

16 $$f_{3aS4} \left( t \right) = A_{1} \beta e^{{ - \left( {\lambda_{1} + \lambda_{{{\text{phys}}}} } \right)t}} + A_{1} \left( {1 - \beta } \right) e^{{ - \left( {\lambda_{{{\text{phys}}}} } \right)t}} \;{\text{with}}\,{\text{shared}}\,{\text{parameter}}\;\beta$$

17 $$f_{3aS5} \left( t \right) = A_{1} \beta e^{{ - \left( {\lambda_{1} + \lambda_{{{\text{phys}}}} } \right)t}} + A_{1} \left( {1 - \beta } \right) e^{{ - \left( {\lambda_{{{\text{phys}}}} } \right)t}} \;{\text{with}}\,{\text{shared}}\,{\text{parameter}}\;A_{1}$$

18 $$f_{3aS6} \left( t \right) = A_{1} \beta e^{{ - \left( {\lambda_{1} + \lambda_{{{\text{phys}}}} } \right)t}} + A_{1} \left( {1 - \beta } \right) e^{{ - \left( {\lambda_{{{\text{phys}}}} } \right)t}} \;{\text{with}}\,{\text{shared}}\,{\text{parameter}}\;\lambda_{1}$$

where parameters $\beta$ are the fractional contributions of the corresponding exponentials with values constrained between 0 and 1. The index $S$ refers to a shared parameter. For completeness, the following exponential functions with one and four estimated parameters were also analysed:

19 $$f_{1} \left( t \right) = A_{1} e^{{ - \lambda_{{{\text{phys}}}} { }t}}$$

20 $$f_{4} \left( t \right) = A_{1} e^{{ - \left( {\lambda_{1} + \lambda_{{{\text{phys}}}} } \right)t}} + A_{2} e^{{ - \left( {\lambda_{2} + \lambda_{{{\text{phys}}}} } \right)t}}$$

### Data fitting

All functions (Eqs. (1)–(20)) were fitted to the biokinetic data of kidneys using the IBMS and the PBMS approaches with all parameters being constrained to positive values. The fittings were performed using the simulation analysis and modelling software SAAMII v.2.3 (The Epsilon Group, Charlottesville, VA, USA) [15]. The following computational settings were used for the fittings: Rosenbrock algorithm, convergence criterion 10^–4^, and absolute-based variance model with a fractional standard deviation of 0.15 [15].

The goodness of the fits were checked by visual inspection of the fitted graphs, the coefficient of variation CV of the fitted parameters (< 0.5) and the off-diagonal values of the correlation matrix (-0.8 < CM < 0.8 for most elements) according to the compilation in Table 1 in Ref. [8].

### Model selection

To select which function is most supported by the data, the corrected Akaike Information Criterion $AICc$, which is corrected for a low ratio of the number of data $N$ to the number of parameters $K$, i.e. *N*/*K* < 40 [11], and the corresponding Akaike weights [11] were calculated as follows:

21 $$AICc = - 2\ln \left( P \right) + 2K + \frac{{2K\left( {K + 1} \right)}}{N - K - 1}$$

22 $$\Delta _{i} = AICc_{i} - AICc_{\min }$$

23 $$w_{{AICc_{i} }} = {{e^{{ - \frac{{\Delta _{i} }}{2}}} } \mathord{\left/ {\vphantom {{e^{{ - \frac{{\Delta _{i} }}{2}}} } { \mathop \sum \limits_{i = i}^{F} e^{{ - \frac{{\Delta _{i} }}{2}}} }}} \right. \kern-\nulldelimiterspace} { \mathop \sum \limits_{i = i}^{F} e^{{ - \frac{{\Delta _{i} }}{2}}} }}$$

where $P$ is the estimated objective function minimized for the fitting, $AICc_{\min }$ is the lowest $AICc$ value of all fitted functions, $\Delta _{i}$ is the difference between the $AICc_{i}$ of function $i$ and $AICc_{\min }$, $F$ is the total number of investigated functions and $w_{{AICc_{i} }}$ is the Akaike weight of function $i$. The Akaike weights indicate the probability that the model is the best among the whole set of considered models [11].

From those functions which passed the goodness-of-fit test (“Data fitting” section), the functions with an Akaike weight > 0.05 were selected as the functions most supported by the data. These were used to determine the area under the curve of the time-activity curve of [^177^Lu]Lu-PSMA-I&T RLT in kidneys.

### Workflow

In the proposed PBMS method, the parameters of Eqs. (1)–(12) were fitted to the kidneys biokinetic data of the population (13 patients). To investigate if the data of the patients could be described by shared parameters, the population fitting was performed to estimate the parameters of functions in Eqs. (13) to (18) with shared parameter estimation. Model selections were performed using the Akaike weights (“Data fitting” section).

In addition to the PBMS method, we also performed the IBMS method [8, 9] using the functions in Eqs. (1)–(12) for patients P1, P3 and P4, for who five biokinetic measurement data points are available. The minimum number of data points for AICc-based model selection is equal to the number of adjustable parameters *K*_max_ + *2* as seen from Eq. (21). Therefore, only for these 3 patients all functions with up to 3 parameters could be used. The best model obtained from the IBMS method of these patients was then used to calculate the TIAs of the [^177^Lu]Lu-PSMA-I&T in all thirteen patients. The performance of the functions selected as most supported by the data using the PBMS and IBMS approach, respectively, was evaluated based on the visual inspection of the fitted graphs. In addition, the relative deviation RD between the TIAs from both approaches was also compared and analysed. The Jackknife method was used to analyse the stability of the best model selected through model selection [11, 16]: For this purpose, the leave-one-out method was applied 13 times with only 12 patients for the calculation of the Akaike weights. The Jackknife was applied to check if the output of the model selection from both PBMS and IBMS would change for different set of data (i.e. leaving one patient out 13 times) used in the analysis.

## Results

Using the PBMS approach, the parameters of the exponential functions in Eqs. (1)–(20) were fitted to the biokinetic data of the kidneys in all patients. The fittings did not pass the goodness-of-fit criteria for 14 of the investigated functions, i.e. the fitting failed based on the visual inspection of the fitted graph or an inadequate goodness of fit (Table 1). Function $f_{4}$ with 4 parameters could not be fitted for patients having data for only 3 time points. From the remaining 5 functions, $f_{3aS4}$ was selected as the function most supported by the data in the PBMS approach based on the Akaike weight of 97% (Table 1). The estimated value of $\beta$, which was fitted as shared parameter in all patients, is (0.9632 ± 0.0037). Based on the Jackknife method, the result of the PBMS method for function $f_{3aS4}$ was very stable (median Akaike weight of 97% with a range of 33%-100%, Table 1).

**Table 1:** Goodness of fits and Akaike weights for the PBMS method

Equation number	Function name	K	Coefficient of Variation CV (max) ^d^	Off-diagonal values of the correlation matrix (max^c^)	Akaike weight (%)	Jackknife Akaike weights (% median [min,max])
1	$f_{2a}$	26	0.04	0.92; 0.95; 0.99^b^	–	–
2	$f_{2b}$	26	0.31	0.81; 0.82; 0.86	0.03	0 [0,50]
3	$f_{2c}$^a^	26	–	–	–	–
4	$f_{2d}$^a^	26	–	–	–	–
5	$f_{2e}$^a^	26	–	–	–	–
6	$f_{3a}$	39	1.96E5^b^	0.93; 0.98; 0.99^b^	–	–
7	$f_{3b}$^a^	39	–	–	–	–
8	$f_{3c}$^a^	39	–	–	–	–
9	$f_{3d}$	39	1.55E + 6^b^	0.95; 0.98; 0.99^b^	–	–
10	$f_{2a,3d}$^a^	26	–	–	–	–
11	$f_{2b, 3d}$^a^	26	–	–	–	–
12	$f_{2c,3d}$^a^	26	–	–	–	–
13	$f_{3aS1}$	26 + 1	4.27E5^b^	0.95; 0.98; 0.99^b^	–	–
14	$f_{3aS2}$	26 + 1	0.32	0.57; 0.59; 0.64	0.04	0 [0,66]
15	$f_{3aS3}$	26 + 1	0.31	0.72; 0.73; 0.76	2.49	3 [0,58]
16	$f_{3aS4}$	26 + 1	0.37	0.79; 0.81; 0.84	97.40	97 [33,100]
17	$f_{3aS5}$	26 + 1	8.64^b^	0.98; 0.99; 1.00^b^	–	–
18	$f_{3aS6}$	26 + 1	0.14	0.64; 0.67; 0.72	0.04	0 [0,3]
19	$f_{1}$^a^	13	–	–	–	–
20	$f_{4}$^e^	52	–	–	–	–

The total number of biokinetic data *N* used in this retrospective analysis is 46, the numbers of parameters of the functions are given in column *K*

^a^The fitting failed based on the visual inspection of the fitted graph

^b^Inadequate goodness of fit (these functions should not be used for model selection)

^c^Three largest (absolute) values of *K* * (*K* − 1)/2 lower-off-diagonal elements. Note that a low percentage of elements only slightly higher than 0.8 is acceptable

^d^CV for the fit parameters calculated as SD divided by the mean

^e^The fitting failed as the number of parameters is larger than the number of data *N* = 46

Using the IBMS approach, the parameters of the exponential functions in Eqs. (1)–(12) were fitted individually to the biokinetic data of kidneys in patients P1, P3 and P4. The goodness-of-fit criteria were not passed for 8 functions (Table 2). Function $f_{2b}$ was selected as the best model in the IBMS approach based on the values of the Akaike weights of 100%, 60% and 100%, for P1, P3 and P4, respectively (Table 2). The Jackknife method was not performed for the IBMS technique because the reduction of the number of data to 4 for patients P1, P3 and P4 allowed the calculation of the AICc weight only for functions with 2 parameters (Eq. (21)).

**Table 2:** AICc values and weights after applying the IBMS method in patients P1, P3 and P4 with biokinetic data of five time points

No	Function	AICc weight (%)^a^		
		P1	P3	P4
1	$f_{2a}$^b^	–	–	–
2	$f_{2b}$	100	60	100
3	$f_{2c}$^b^	–	–	–
4	$f_{2d}$^b^	–	–	–
5	$f_{2e}$^b^	–	–	–
6	$f_{3a}$	0	40	0
7	$f_{3b}$^c^	–	–	–
8	$f_{3c}$	0	0	0
9	$f_{3d}$^c^	–	–	–
10	$f_{2a,3d}$	–	–	–
11	$f_{2b,3d}$^b^	–	–	–
12	$f_{2c,3d}$^b^	–	–	–

Equations (13)–(18) with shared parameters, which are designed for PBMS, were not included in the IBMS analysis. Function (19) failed based on visual inspection. For function (20) AICc could not be calculated as there are 4 fit parameters for only 5 data (compare Eq. (21))

^a^All the zeros stand for values lower than 10^–5^

^b^The fitting failed based on visual inspection of the graph

^c^Inadequate goodness of fit (these functions should not be used for model selection)

**Fig. 1:**
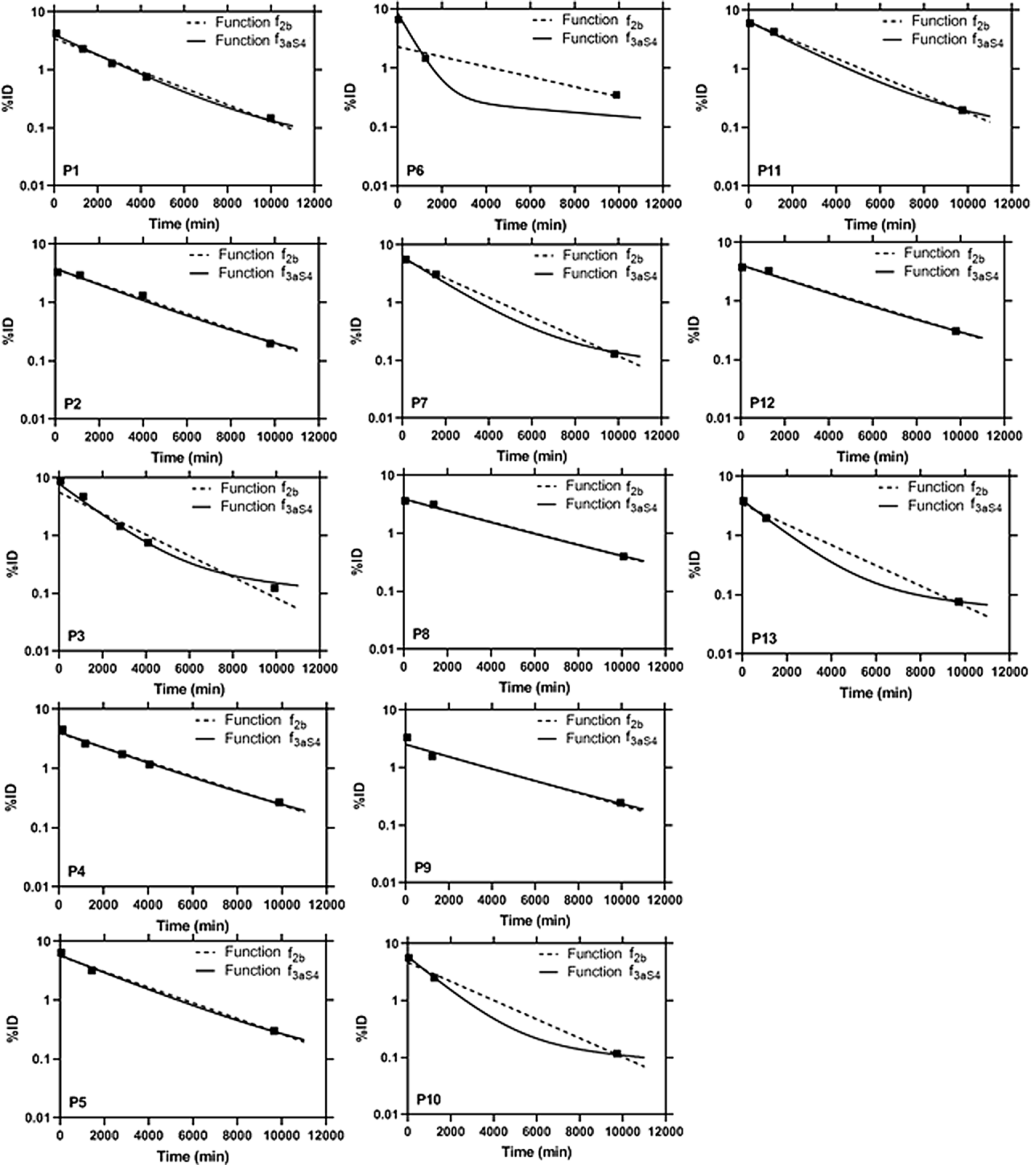
Time-Activity data and fit curves of the two functions most supported by the data, $f_{3aS4}$ and $f_{2b}$, which were derived using the PBMS and IBMS method, respectively

**Fig. 2:**
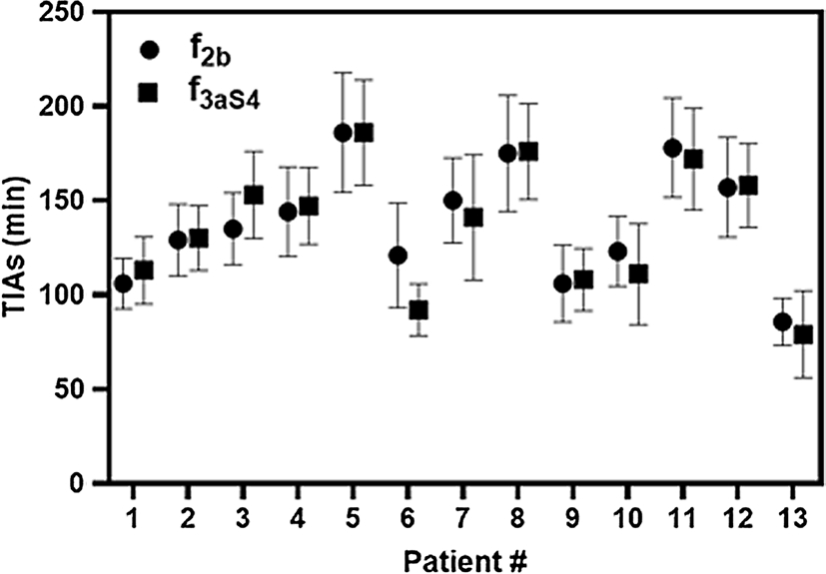
Kidneys TIAs calculated from the two functions most supported by the data, $f_{3aS4}$ and $f_{2b}$, which were derived using the PBMS and IBMS method, respectively

Figure 1 shows the comparison of function $f_{3aS4}$ obtained from the PBMS approach and function $f_{2b}$ from the IBMS approach in explaining the investigated biokinetic data of kidneys. Visual inspection of the graphs in Fig. 1 shows that function $f_{3aS4}$ has a relatively better or at least equivalent performance as function $f_{2b}$. Figure 2 presents the corresponding time-integrated activities (TIAs).

## Discussion

In this work, we applied population-based model selection to calculate individual TIAs, the accurate determination of which is important for individual dosimetry and treatment planning. The use of a model selection procedure is advantageous, because it increases the reproducibility of results by objectively selecting a fit function from a set of functions (models), in contrast to the application of rule of thumbs [7] or simply user-guessing. The selection of a good mathematical model (i.e. function) for the calculation of TIAs is important, as using of an improper function will invalidate or at least deteriorate the result. Therefore, model selection is an important and critical aspect of scientific data analysis [12].

Available population data in nuclear medicine are usually heterogeneous and sparse. The presented method can be used for this common situation. Pharmacokinetic information of heterogeneous data can be derived from a population and introduced for the individual fit. The advantages of our method are achieved by improving both inputs, i.e. (1) the data and (2) the set of models from which the best one is selected. This in turn also improves the result.

1. Data of a population instead of just a single patient are used for the model selection procedure. In [^177^Lu]Lu-PSMA-I&T radioligand therapy as our example, the use of the $f_{2b}$ function is the case for which both the PBMS and IBMS approaches are identical. As seen from Table 1, the Akaike weight, i.e. the probability for $f_{2b}$ to be the best function, is lower by a factor of larger than 3247 compared to the $f_{3aS4}$ function, which indicates significantly better fits. Also, $f_{2b}$ is the function with the lowest probability of all functions with an acceptable goodness of fit. Assessing the stability of the model selection procedure needs the application of the Jackknife method [11, 16]: For the PBMS best function $f_{3aS4}$, removing one patient having 5, 4 or 3 data points results in *N/K* ratios of 41/25≈1.64, 42/25≈1.68, 43/25≈1.72, respectively. These ratios differ only slightly from those of the total patient population: *N*/*K* = 46/27 ≈ 1.70. For the IBMS best function $f_{2a}$, one data point of the patient under consideration must be removed for the stability assessment. Thus, removing one data point for patients having 5, 4 or 3 data points results in *N*/*K* ratios of 4/2 = 2, 3/2 = 1.5, 2/2 = 1, respectively. However, from Eq. (21), it follows for the calculation of AICc that *K*_*max*_ = *N* *−* 2. Thus, assessing the stability of the IBMS method becomes impossible for patients having only 4 or 3 data points and most likely unstable for patients with 5 available data points.
  This higher stability of the PBMS results compared to the IBMS is also seen when comparing the results in Tables 1 and 2: Whereas for the PBMS method the Akaike weight for the best function is 97.4% (Table 1) with a median of 97% and a range from 33 to 100%, for the IBMS method the best fit function of one patient (P3, Table 2) is quite uncertain with a weight of only 60% and—most importantly—the Jackknife method to calculate an uncertainty for the Akaike weights is impossible for all three patients.
2. The set of models, from which the best one is selected, is also constrained by *K*_max_ = (*N* *−* 2) [8, 9, 11, 12]. Therefore, in our example, the PBMS method in principle would allow to include in the model set functions with up to 44 parameters. Clearly, more and more complex functions in the function permit a better model selection result and thus also better reflect the true biokinetics. In contrast, individual model selection (e.g. for patients with three data points) is possible only for functions depending on only one parameter. Such functions will however not be able to adequately reflect the biokinetics.

Another advantage of the PMBS method is the possibility to use functions with shared parameters in the population. For our patient population, we yielded function $f_{3aS4} \left( t \right) = A_{1} \beta e^{{ - \left( {\lambda_{1} + \lambda_{{{\text{phys}}}} } \right)t}} + A_{1} \left( {1 - \beta } \right) e^{{ - \left( {\lambda_{{{\text{phys}}}} } \right)t}}$ for the estimation of kidneys TIAs with $\beta$ = 0.9632. This result can be applied to future patients by using the shared parameter as fixed parameter and estimate $A_{1}$ and $\lambda_{1}$ only for the subsequent patients. Thus, once the best model has been identified, this model can be used for subsequent patients with corresponding fixed parameters. Even patients having less data can be fitted using such shared parameters as fixed parameters.

A general problem in clinical dosimetry is that it is unclear which function to fit to the data. This is even true for cases with many data per organ, but even more relevant for those cases with only few data. This is also a reproducibility issue, as every user will possibly use another function yielding very different results. Our proposed method however will be much more reproducible due to two reasons: first, we use many functions and select the best (model selection): This already reduces variability in results obtained by different users. Second, also model selection has an uncertainty, which may be even impossible to calculate as we show for the IBMS in our example if applied to the data of only one patient. This uncertainty is greatly reduced in the PBMS approach by adding the information contained in the population of similar patients (Tables 1, 2).

For our example, we can clearly see from the graphs in Fig. 1 that function $f_{3aS4}$ obtained from the PBMS approach has a better or similar performance compared to function $f_{2b}$ which is preferred by the IBMS approach. Figure 2 demonstrates the large effect the chosen fit function may have for the TIAs of some patients (e.g. P6).

The great advantages of using PBMS over IBMS presuppose that the kinetics in the population have appropriate commonalities that are correctly detected by PBMS. For this, it is particularly necessary to include the "correct" functions in the set of functions examined. For example, if we had not included function $f_{3aS4}$ in our set of functions, function $f_{3aS3}$ would have been selected as the best function with an Akaike weight of 98.1% (which is even higher than for function $f_{3aS4}$). Therefore, based on the Akaike weight alone, we cannot already conclude that a function is very good. Consequently, great emphasis must be placed on including all relevant functions in the model set.

Biokinetic data of kidneys in [^177^Lu]Lu-PSMA-I&T radioligand therapy were used to demonstrate the procedure. However, the method can be used and implemented for different organs and also for tumours. The only part of the procedure that maybe needs to be adapted to different organs relates to the set of functions, as this set should contain suitable functions that can well describe the biokinetics of the organ in consideration. For example, if the tumours have a long accumulation phase, one would need to include appropriate functions in the function set so that then the function best supported by the data actually describes the corresponding biokinetics well.

Input, processing and output of the proposed PBMS method in this study have the following limitations.

For the input of the PBMS:

1. The uncertainty of the quantitative data might affect the model selection. It has been shown that accurate and precise quantitative data are essential as input for the calculation of TIAs [17] and the “garbage in-garbage out” principles applies. As the main purpose of this study is to introduce the PBMS method, implementation of the method for certain cases, e.g. implementation in different organs or analysing the effect of image quantification, is beyond the scope of this study.
2. The number of data used in our study is relatively low. In this paper, we present a method that is mainly needed in cases where not many data are available. Therefore, it is consequentially important that the method is presented for patients with only limited data. Of course, more data would lead to more accurate and precise results. Although the low number of data naturally limits the significance of our results, on the other hand, the results show that our approach works.
3. The model selection used in our study is based on a specific method, i.e. the AICc. There exist also other methods for model selections such as the F-Test [11, 18] and the Bayesian Information Criterion (BIC, [11, 19]). However, the AICc method has been shown to be an effective and efficient approach, applicable to nested and non-nested models [11].
4. Sums of exponential functions with increasing complexity were used in the investigated model set, as such mathematical functions are commonly used to describe biological processes [6–9]. In all functions, the physical decay is implemented as a factor, as it was shown that such an approach yields better results if $\lambda_{i} \ge 0$ is additionally assumed [20, 21]. There are, however, no general rules which functions to include in the set of model functions, except that one should use all available theoretical and empirical information to define an adequate set of candidate models a priori [11]. This is a consequence of the AICc only being able to select the Kullback–Leibler best model from the candidate models. “If all candidate models are poor, the AICc will select the best approximating, but nevertheless poor model”. [11].
  Additional (non-exponential) functions could have been added to the set of the tested functions in our analysis. This is however not supported by prior empirical knowledge as exponential functions are sufficient to describe most biokinetics. Based on the biokinetic data presented in Figure 1, also the addition of sums of exponentials with more parameters will not be effective. Although we could gain higher confidence in the results of our model selection by testing a larger number of functions, this will increase the workload giving most likely the same result for the function that is best supported by the data.
5. In this study, we propose a method based only on available data. Clearly, the investigation of the effect of different time schedules on the improvement when using this method would also be of interest, but is beyond the scope of this study.

For the processing of the PMBS: Certain software, i.e. SAAMII, was used for the fitting analysis. However, in order that a fit is reproducible, the same input data, the same objective function and an arbitrary algorithm, which will find the minimum of the objective function, is sufficient. Therefore, any software being capable of such an algorithm will yield the same results. A software that uses the same algorithm is, for example, the NUKFIT software [8], which is free for academic use.

For the output of the PBMS: In the worst case, the population information contained in the best function derived using the PBMS method may not be suitable for the accurate determination of the TIA of the subsequent patient. However, this is unlikely to happen as it has been shown in many studies that the implementations of population information could improve the accuracy of TIAs calculation [3, 22].

## Conclusions

In this retrospective analysis, we propose a method for performing a model selection for a patient population to estimate individual TIAs for subsequent patients. By using the proposed method, we can obtain a better justified function for the determination of TIAs, as the model selection is based on a patient population, i.e. on more data, instead of only on one patient. More data, on one hand, allow a higher number of parameters of the investigated fit functions and thus increase the space of fit functions that can be included in the set of functions for model selection. On the other hand, it reduces the uncertainty of the obtained Akaike weights and thus the uncertainty in the selected most supported fit function. This approach is especially important if—as is often the case in clinical nuclear medicine—only a low number of biokinetic data per patient is available in the patient population under consideration.

## Data Availability

The used data are available from the corresponding author on reasonable request.

## Abbreviations

AICc: corrected Akaike information criterion
IBMS: Individual-based model selection
PBMS: Population-based model selection
RD: Relative deviation
RLT: Radioligand therapy
TIA: Time-integrated activity
